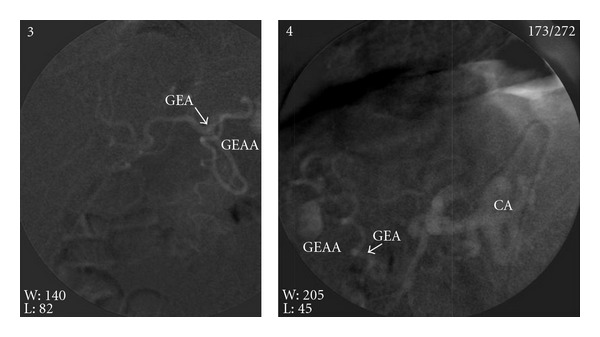# An Early Diagnosis of Gastroepiploic Arterial Aneurysm during a Routine Abdominal Ultrasound Study

**DOI:** 10.1155/2011/570239

**Published:** 2011-07-28

**Authors:** Giovanni Casella, Adriana Sartirana, Pietro Vandoni, Camillo Di Bella, Fabio Pagni, Paolo Nobili

**Affiliations:** ^1^Medical Department, Desio Hospital, Piazza Benefattori, Monza e Brianza, 1 20033 Desio, Italy; ^2^Radiology Department, Desio Hospital, Desio, Monza e Brianza, Italy; ^3^Cardiology Department, Desio Hospital, Desio, Monza e Brianza, Italy; ^4^Pathology Department, Desio Hospital, Desio, Monza e Brianza, Italy; ^5^Vascular Surgery Department, Clinica “San Giuseppe”, Milan, Italy

## Abstract

Gastroepiploic arterial aneurysm (GEAA) is a rare condition, but the rupture risk is very high. We report the case of a patient with incidental finding of GEAA during US examination. The diagnosis was confirmed by a computed tomography and an angiographic study. A classic laparotomy with aneurysmectomy has been successfully performed.

## 1. Case Report

Gastroepiploic arterial aneurysm (GEAA) is a rare condition, but the rupture risk is very high [1] because in 90% of the affected patients it is shown as a vascular emergency [1]. Visceral arterial aneurysms are rare and their incidence in the general population has been reported at 0.01–2% [2]. We describe a diagnosis of unruptured gastroepiploic artery aneurysm by (thank) a routine ultrasound diagnosis. We observed, in June 2005, a-64-year old Caucasian man, HbsAg “carrier” with consecutively normal liver enzymes. During a routine ultrasound examination, we noted an abnormal circular anechogenic area of 2.5 cm in diameter in the epigastric region, near the visceral peritoneal layer. A Doppler study revealed an arterial pulse suggesting the hypothesis of an arterial aneurismatic dilation. This hypothesis has been confirmed by a computed tomography (CT) and an angiographic study (Figure 1), and we concluded for right GEAA without evidence of other splachnic aneurysms. Non-surgical treatment as transcatheter arterial embolization (TAE) has been considered, but it was impossible to perform for a long and tortuous vessel's anatomy. A classic laparotomy with aneurysmectomy has been successfully performed. Histologic examination and absence of blood-antineutrophil Cytoplasm antibodies (P-ANCA) ruled out polyarteritis nodosa (PAN). The patient, at present after 5 years, is in good general condition. Stanley and Zelenoch [2] reported a study about more than 3000 visceral arterial aneurysms [2]- (this data is confirmed); 60% are splenic, 20% of the hepatic artery, 5.5% of the (mesenteric) superior mesenteric artery, 4% of the celiac axis, 2% of the pancreatic duodenal artery and its branches, 1.5% of the gastroduodenal artery, and less than 1% of the inferior mesenteric artery. Pulli et al. [3], in a series of 55 patients affected by visceral artery aneurysm, reported only 1 case of right gastroepiploic aneurysm (1.7%). These GAAs are mostly common in 50–60 years old men, often affected by arterial hypertension and atherosclerosis [2]. Other etiologic factors are infection, medial necrosis, trauma, pregnancy, portal hypertension, biliary disease, pancreatitis, and connective tissue disease [2]. Clinical manifestations of gastrointestinal arterial aneurysmes (GAA) vary from free of symptoms to epigastric pain. Pulli et al. [3] reported that the diagnosis was incidentally made during ultrasound examination performed for unrelated abdominal disease in 52 of all 55 patients with visceral artery aneurysm (94.6%) [3]. Up to 50% of patients remain asymptomatic before the aneurysm rupture and operative mortality is 50–70% for emergency procedures versus 0–3% for elective surgery [3]. The widespread use of “Imaging” devices permits an early diagnosis in asymptomatic patients allowing an “elective” surgical therapy [3]. About 81% of the GEAAs are diagnosed after the rupture [4]. The mortality of these aneurysms after rupture is very high [4]; aneurysmectomy is mandatory if the diagnosis has been performed before the rupture [4]. Angiography should be performed in all patients with single aneurysm because 15–40% of these have multiple aneurysms as the cases reported by Pulli et al. [3] that showed multiple visceral artery aneurysm (VAA). Borioni et al. [4] reported that this can be eliminated. Celiac arteriography (CA) permits a correct diagnosis in 44.4% of all patients studied, surgery in 50% of the patients, and CT with medium contrast only in 5.6%. This low diagnostic level of CA could be due to the slow flow of blood in this tract that it cannot be visualized by CA [4]. Surgical excision may be performed by traditional surgery or by laparoscopic surgery [4]. Recently, trancatheter arterial embolization (TAE) -no (TACE) with an occlusion of the aneurysm should be always considered before every surgical procedure [5].

## Figures and Tables

**Figure 1:**